# DNA Replication Timing Is Maintained Genome-Wide in Primary Human Myoblasts Independent of D4Z4 Contraction in FSH Muscular Dystrophy

**DOI:** 10.1371/journal.pone.0027413

**Published:** 2011-11-11

**Authors:** Benjamin D. Pope, Koji Tsumagari, Dana Battaglia, Tyrone Ryba, Ichiro Hiratani, Melanie Ehrlich, David M. Gilbert

**Affiliations:** 1 Department of Biological Science, Florida State University, Tallahassee, Florida, United States of America; 2 Human Genetics Program, Department of Biochemistry, and Tulane Cancer Center, Tulane Medical School, New Orleans, Louisiana, United States of America; University Hospital Vall d'Hebron, Spain

## Abstract

Facioscapulohumeral muscular dystrophy (FSHD) is linked to contraction of an array of tandem 3.3-kb repeats (D4Z4) at 4q35.2 from 11-100 copies to 1-10 copies. The extent to which D4Z4 contraction at 4q35.2 affects overall 4q35.2 chromatin organization remains unclear. Because DNA replication timing is highly predictive of long-range chromatin interactions, we generated genome-wide replication-timing profiles for FSHD and control myogenic precursor cells. We compared non-immortalized myoblasts from four FSHD patients and three control individuals to each other and to a variety of other human cell types. This study also represents the first genome-wide comparison of replication timing profiles in non-immortalized human cell cultures. Myoblasts from both control and FSHD individuals all shared a myoblast-specific replication profile. In contrast, male and female individuals were readily distinguished by monoallelic differences in replication timing at DXZ4 and other regions across the X chromosome affected by X inactivation. We conclude that replication timing is a robust cell-type specific feature that is unaffected by FSHD-related D4Z4 contraction.

## Introduction

Facioscapulohumeral muscular dystrophy (FSHD) is an autosomal dominant genetic disorder characterized by progressive muscle weakness and wasting that typically initiates in the face, shoulder-girdle and upper arm. Together with clinical characteristics of the disease, transcription-profiling studies support a model for FSHD involving impaired muscle regeneration [1], [2]. FSHD is linked to contraction of the D4Z4 tandem repeat at subtelomeric 4q35.2 (OMIM 606009). Usually 11-100 copies of the 3.3 kb repeat unit are non-pathogenic while 1-10 copies correlate with onset of FSHD in 95% of patients [3]. Another D4Z4 macrosatellite array highly homologous to that of 4q35.2 is present at 10q26.3, yet D4Z4 contractions at 10q26 are almost never pathogenic [4]. The current paradigm suggests that pathogenicity of D4Z4 at 4q35.2 is linked to FSHD through a D4Z4 gene encoding Double Homeobox Protein 4 (*DUX4*) [5], [4], [6]; however, its pathogenic mechanism is unclear. The open reading frame encoding DUX4 protein within each D4Z4 repeat unit lacks a consensus signal for polyadenylation [7]. A single nucleotide polymorphism distal to the last D4Z4 repeat is found in all FSHD patients and stabilizes *DUX4* transcripts by providing a polyadenylation signal and thereby creating a toxic gain-of-function mutation [4], [8]. However, although forced *DUX4* expression inhibits myogenesis and decreases *Myogenic Differentiation 1* (*MYOD1*) transcription [9], [10], only about one in a thousand FSHD myoblasts has detectable *DUX4* expression and expression in FSHD myotubes is not much stronger [6], [2]. In addition, the polyadenylation signal polymorphism, which stabilizes the DUX4-encoding transcript is prevalent in the general population and, therefore, disease status is still strongly linked to D4Z4 contraction per se. We recently proposed a model in which frequent, but transient, expression of *DUX4* at a pre-myoblast stage drives the muscular dystrophy phenotype of FSHD patients [2].

While DUX4 involvement in FSHD pathogenesis clearly requires at least one D4Z4 repeat, the mechanism by which D4Z4 contraction beyond a threshold array length usually leads to *DUX4* expression remains uncertain. It has been proposed that the repetitive nature of D4Z4 arrays creates a heterochromatin environment at 4q35.2 that maintains low regional gene expression under normal conditions [11], [12], and that the loss of this heterochromatic region is pathogenic. Several studies identified hallmarks of heterochromatin in normal individuals at the D4Z4 region and at a similar macrosatellite repeat on the human X chromosome called DXZ4 [13]-[16]. Although evidence of chromatin relaxation has been observed in D4Z4 at 4q35.2 in FSHD patients with contracted D4Z4 arrays, rare cases of FSHD patients without contraction of the D4Z4 array at 4q35.2 also display chromatin relaxation [14]–[16]. Moreover, epigenetic marks indicative of heterochromatin do not appear to spread from full length or contracted D4Z4 repeats [17], [18] and microarray studies have not detected any gradient of altered gene expression in the 4q35.2 region [1], [2], [19]. As an alternative hypothesis, it has been proposed that long-range chromatin interactions distant from the 4q35.2 D4Z4 locus may occur in the muscle lineage as a result of pathogenic contraction of the D4Z4 array [15], [17], [18], [20]–[22].

The temporal order of replication of chromosomal segments is reflective of cell-type-specific chromatin organization and changes coordinately with the differentiation state during development [23]–[29]. We recently demonstrated that genome-wide maps of long-range chromatin interactions generated by high-resolution chromatin conformation capture methods (Hi-C maps) [30], can be almost precisely mirrored by genome-wide profiles of replication timing [27]. Since replication-timing profiles are much easier to generate than Hi-C maps, we examined whether replication-timing maps could provide evidence for the existence of novel long-range chromatin interactions in myoblasts from FSHD versus control individuals. Although the replication timing in the D4Z4 vicinity at 4q35.2 is unperturbed in FSHD myoblasts [31], it remained possible that array contraction could alter chromosome folding at long distances from the repeat. Indeed, artificially seeded telomeres were shown to replicate later when adjacent to a single D4Z4 repeat than when adjacent to multiple repeats [32]. Additionally, since even closely related cell types can be clearly distinguished by comparing genome-wide replication timing profiles [26], the hypothesis that muscle differentiation is altered in FSHD [1], [2] suggests that at least some replication timing profile differences might exist between control and FSHD myoblasts. We found that replication timing profiles from control myoblasts were indistinguishable from those obtained from FSHD myoblasts. The maintenance of replication timing profiles among human myoblast cultures derived from different healthy muscle biopsies or from a disease background underscores the robustness of the replication timing program within a given cell type. In addition, the lack of replication timing differences genome-wide suggest that D4Z4 chromatin relaxation at 4q35 does not cause irregular long-range chromatin interactions in FSHD myogenic precursors.

## Results and Discussion

### FSHD and control myoblast cultures

In studies of primary myoblasts, it is critical to determine what percentage of the cells are actually myoblasts because contaminating fibroblast-like cells can have a growth advantage if optimal conditions are not maintained for the myoblasts. We showed that >85% of the cells in aliquots from all batches of FSHD and control myoblasts used in this study were myoblasts by immunostaining with desmin, a muscle-specific marker not expressed in fibroblasts. In addition, we verified that these batches of cells could efficiently differentiate to myotubes (multinucleated, desmin-immunopositive, and heavy chain myosin-immunopositive). Similarly, Winokur et al. and Barro et al. [33], [34] showed that FSHD and control myoblasts form multinucleated myotubes with equal efficiency.

Because full-length *DUX4* transcripts from the pathogenic, contracted D4Z4 repeat array at 4q35 are associated with FSHD, we assayed, by previously described methods [4], six control and six FSHD myoblast samples, including three samples used in the present study [2]. We found, as previously reported [6], that the average levels of these transcripts were extremely low in FSHD myoblasts, that this transcript was undetectable in control myoblasts, and that only some of the FSHD myoblast cell strains had detectable transcripts [2]. The control samples CM4 and CM5, used in the present study, were negative and FM7, used in this study, was positive for these transcripts under RT-PCR conditions previously reported (400 ng of cDNA per assay and qRT-PCR conditions [4]). Because only about 1/1000 FSHD myoblasts produce detectable full-length *DUX4* transcripts [6], the presence or absence of these transcripts should not detectably influence the behavior of a culture of FSHD myoblasts. Nonetheless, well-characterized FSHD myoblast cultures have an oxidative stress hypersensitivity phenotype [33], [34] and, as we have recently shown, a highly significantly dysregulated expression profile [2]. Therefore, we tested these cells from biopsies of moderately affected muscle of FSHD patients for irregularities in DNA replication timing compared to normal-control myoblasts.

### FSHD and control myoblasts share a common genome-wide replication timing profile

**Figure 1:**
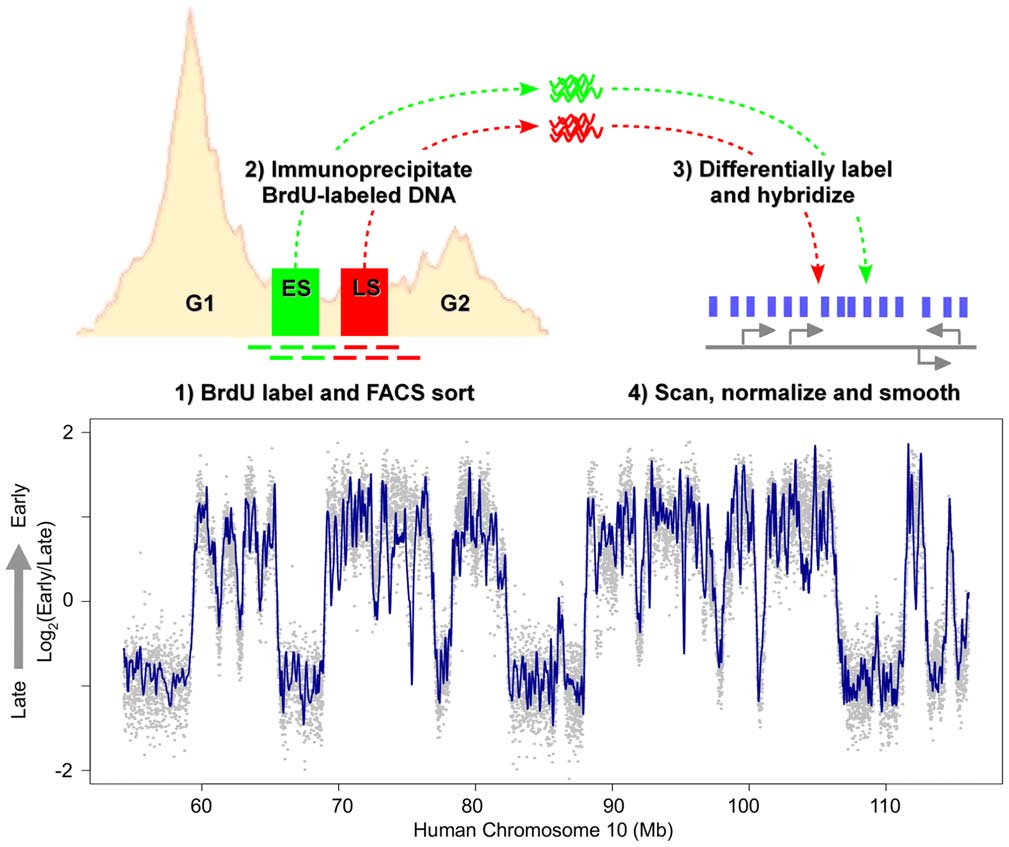
Method for generation of genome-wide replication profiles of primary myoblasts. Step 1 illustrates BrdU labeling of actively dividing cells for 2 hours, followed by fixation and FACS sorting by DNA content into early and late S phase fractions. In step 2, BrdU-labeled DNA from each fraction was isolated by immunoprecipitation. Step 3 involved differential-labeling and co-hybridization to a whole-genome human CGH microarray. For the final step, arrays were scanned and the extracted log_2_(early/late) raw data was Loess normalized (represented by gray data points) and then smoothed (dark blue curve) [35]. Shown is a profile of approximately 60 Mb of chromosome 10 for patient CM1.

**Figure 2:**
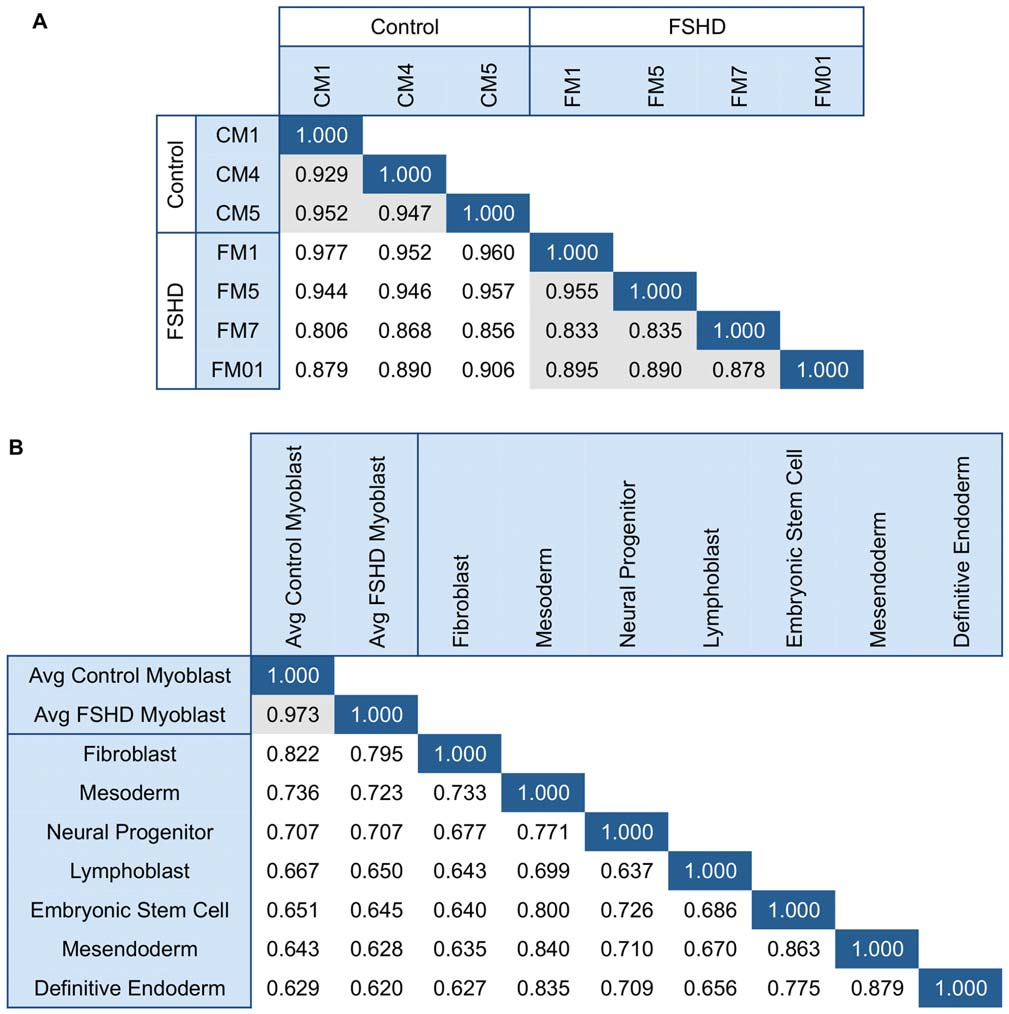
Similarity between FSHD and control myoblast genome-wide replication timing profiles and other cell types. (A) Genome-wide Pearson's correlations between individual control (begins with “C”) and FSHD (begins with “F”) primary myoblast timing profiles are displayed. Gray indicates control to control or FSHD to FSHD comparison. One FSHD dataset (FM7) had a low signal to noise ratio and deviates somewhat from other datasets, but was still included for comparison. (B) Genome-wide Pearson's correlations between averaged control and FSHD myoblasts as well as other human cell types are displayed. Gray indicates control to FSHD myoblast comparison.

To determine the extent to which the FSHD disease background affects the replication timing program, we profiled replication timing in FSHD and control primary myoblasts genome-wide. Generation of genome-wide replication timing profiles is illustrated in Figure 1. First, nascent DNA in asynchronously growing myoblasts was labeled with 5-bromo-2-deoxyuridine (BrdU). Labeled myoblasts were then sorted into early and late S-phase fractions based on DNA content using flow cytometry, and BrdU-labeled DNA was purified by immunoprecipitation. Purified DNA fractions were differentially labeled and co-hybridized to a Comparative Genomic Hybridization (CGH) microarray with 2.5 kb median probe spacing across the entire human genome. Hybridization data was then Loess normalized and smoothed to provide a genome-wide profile with relative replication timing values for each probe position [35]. Similar Pearson's correlations resulted when genome-wide myoblast replication timing profiles from three control individuals were compared to each other and to myoblast profiles from four FSHD individuals (Figure 2A). Biological replicates from different cell lines using this genome-wide replication timing method routinely show high correlation to each other [27] and are consistent with profiles created at higher probe density [23] or by deep sequencing of similarly prepared BrdU-labeled nascent strands [27]. Thus, the high genome-wide correlations indicate the replication-timing program is largely maintained in the FSHD myoblasts profiled.

### D4Z4 repeat contraction does not affect 4q35 or 10q26 replication timing

**Figure 3:**
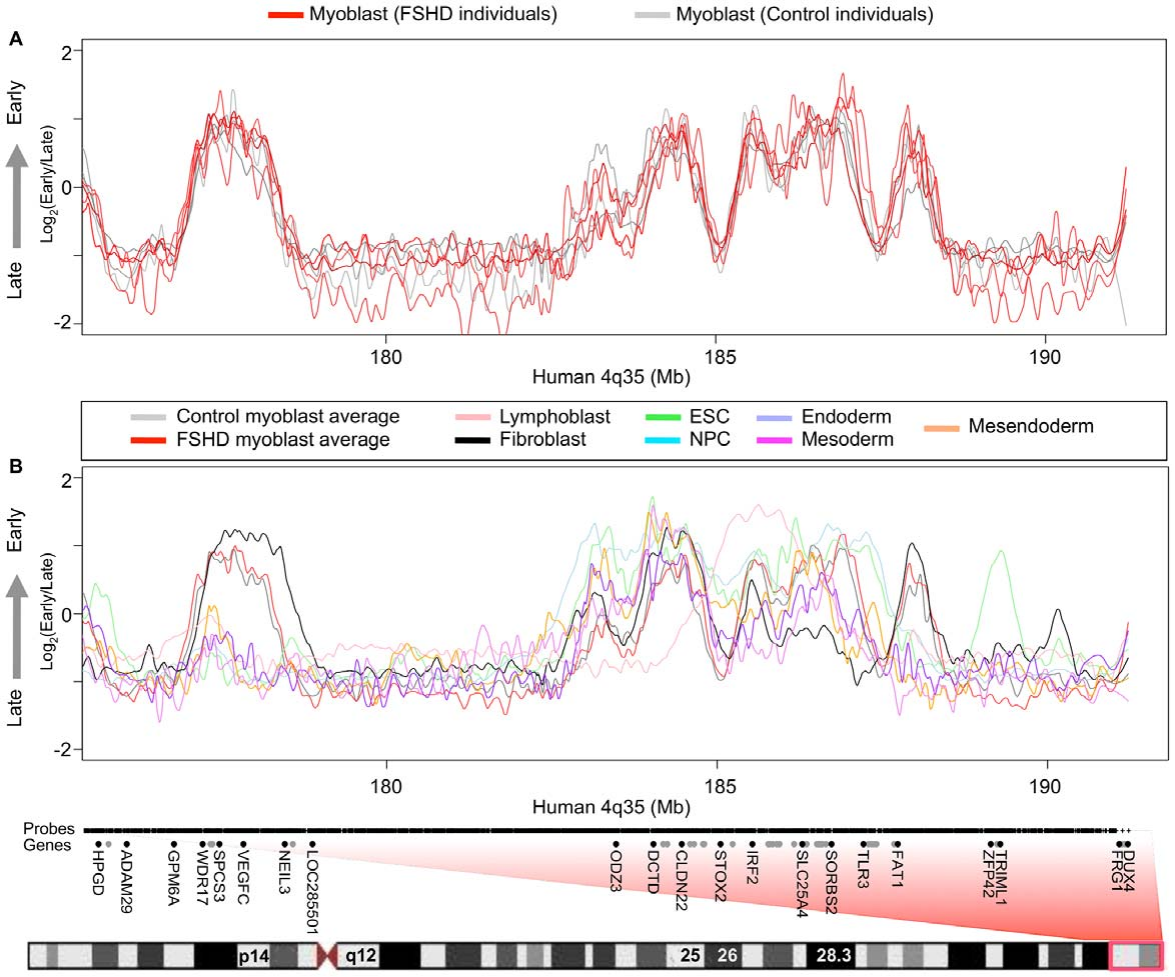
D4Z4 contraction and FSHD disease background do not affect 4q35 replication timing. (A) Primary human myoblast replication timing profiles are shown for 3 control (gray) individuals and 4 FSHD (red) patients across the proximal region to 4q35. One FSHD dataset (FM7) had a low signal to noise ratio and deviates somewhat from other datasets, but was still included for comparison. (B) Replication timing profiles for many different human cell types are shown across the proximal region to 4q35. Boxed in red is the region of human chromosome 4 corresponding to 4q35. Microarray probe positions are indicated along the X-axis by short black vertical lines. Genes are indicated by black dots, for those whose name is given, or gray dots, for those with too high a density to add a label. *DUX4* transcripts derived from 4q35.2 D4Z4 map between hg18 chromosome 4 coordinates 191,229,361 and 191,247,457 (UCSC Genome Browser, http://genome.ucsc.edu/).

**Figure 4:**
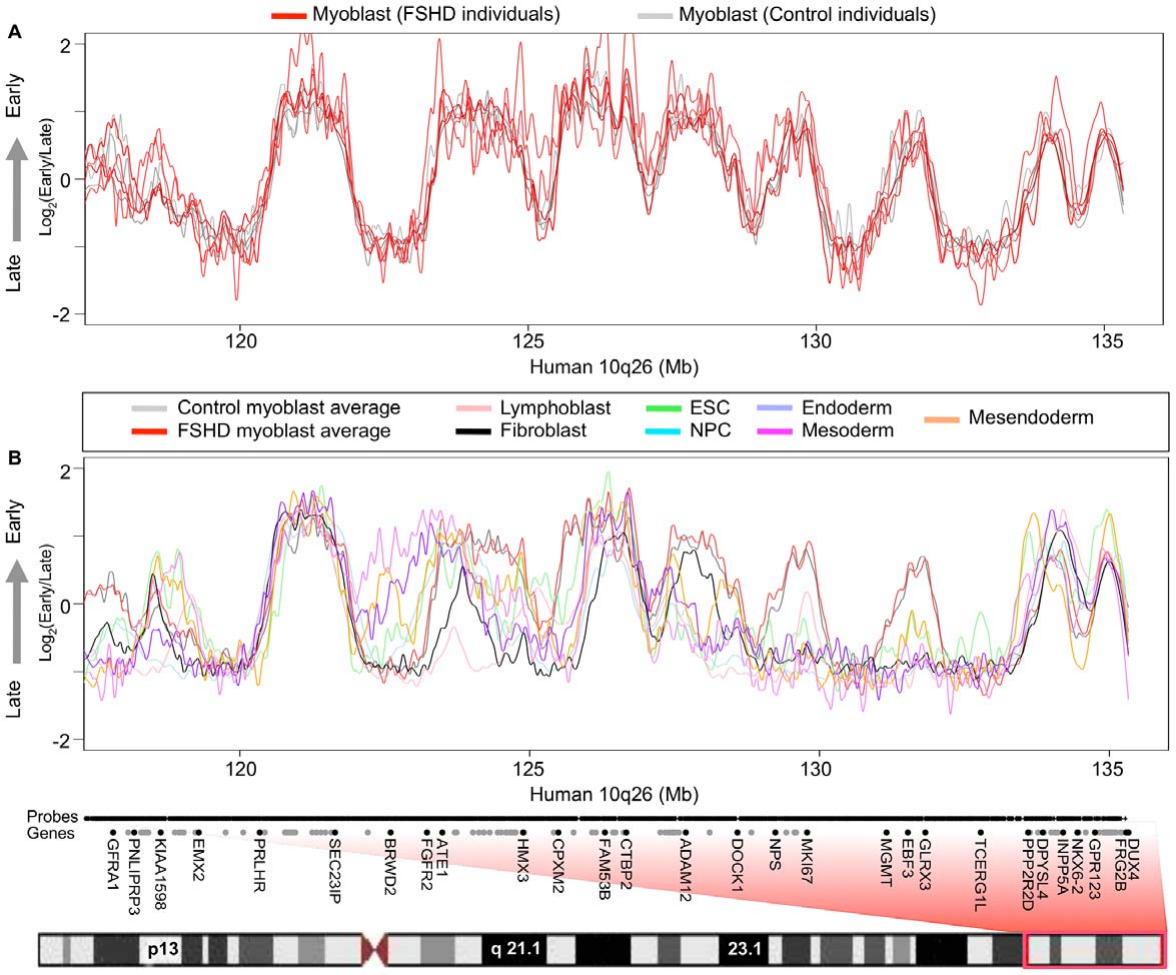
D4Z4 contraction and FSHD disease background do not affect 10q26 replication timing. (A) Primary human myoblast replication timing profiles are shown for 3 control (gray) individuals and 4 FSHD (red) patients across the proximal region to 10q26. One FSHD dataset (FM7) had a low signal to noise ratio and deviates somewhat from other datasets, but was still included for comparison. (B) Replication timing profiles for many different human cell types are shown across the proximal region to 10q26. Boxed in red is the region of human chromosome 10 corresponding to 10q26. Microarray probe positions are indicated along the X-axis by short black vertical lines. Genes are indicated by black dots, for those whose name is given, or gray dots, for those with too high a density to add a label. DUX4 transcripts derived from 10q26.3 D4Z4 map between hg18 chromosome 10 coordinates 135,330,358 and 135,338,574 (UCSC Genome Browser, http://genome.ucsc.edu/).

Three groups found no detectable difference in the intranuclear localization of unique probes in the vicinity of D4Z4 at 4q35.2 in FSHD versus control myoblasts [12], [31], [36]. However, there are several reports of evidence for changes in chromatin organization at 4q35.2 within the 200-kb region distal to D4Z4 that contains mostly segmental duplications [21], [22]. To identify any localized changes proximal to the subtelomeric D4Z4 arrays, we more closely examined FSHD-linked 4q35 and highly homologous 10q26 subtelomeres, which contain almost identical D4Z4 repeat units. All the FSHD myoblast samples profiled came from patients with documented contraction of a 4q35 D4Z4 array. The control samples were obtained from unaffected first-degree relatives of FSHD patients over the age of 25, and most patients exhibit symptoms in their teenage years [3]. Therefore, because FSHD is a dominant genetic disease, the controls are expected to contain 4q35 D4Z4 arrays of normal size as was verified for one of the control samples (CM1). We found that replication timing was indistinguishable in FSHD myoblasts relative to control myoblasts at both 4q35 (Figure 3A) and 10q26 (Figure 4A). Due to the repetitive nature of D4Z4 and its location in a region of segmental duplication, the closest unique probes on our microarrays were about 100 kb proximal to 4q35.2 D4Z4 and 45 kb proximal to 10q26.3. Nonetheless, replication forks move at approximately 2 kb per minute, so that at least 200 kb of DNA is labeled during the 2-hour BrdU labeling period [35]. Hence, any replication timing changes in the D4Z4 repeat should be readily detected 45 kb away. Moreover, a previous study using fluorescence *in situ* hybridization concluded that D4Z4 repeat contraction has no local effect on replication timing [31].

### Replication timing profiles reflect efficient myoblast differentiation in FSHD

Because FSHD was proposed to be a disease involving muscle differentiation [1], we compared our genome-wide myoblast profiles to those from various human cell types. The replication-timing program is extensively re-organized during differentiation of all cell types examined to date [23]–[29], and developmental changes in replication timing reflect concomitant changes in large-scale chromatin organization [20], [37]. We found that averaged profiles for FSHD and control myoblasts were significantly more similar to each other genome-wide than would be expected for profiles from different cell types (Figure 2B). Close examination of both 4q35 and 10q26 reveals overall myoblast-specific replication timing profiles with several individual regions that are replicated at different times in different cell types (Figures 3B and 4B). *SLC25A4*, for example, is an FSHD-candidate gene on 4q35.1 that replicates early in some cell types (including myoblasts) but replicates in mid or late S phase in other cell types. Further, the 4q35.2 cluster of genes *ZFP42, TRIML1, and TRIML2,* and the surrounding gene desert replicated early in embryonic stem cells (ESCs) and late in all other cell types consistent with the association of these genes with early embryogenesis (Figure 3B). *FAT1* replicated earlier during S phase in myoblasts and fibroblasts, which strongly transcribe this gene [2]. Additionally, a second gene desert proximal to *FRG1* and distal to *TRIML1* contains a region that replicates early specifically in fibroblasts, while *FRG1* itself replicates in mid to late S phase in all cell types examined. Interestingly, the *FRG1*-proximal gene desert contains a DNaseI hypersensitive site found in fibroblasts and observed preferentially in FSHD versus control myoblasts [38]. However, replication-timing profiles were indistinguishable between FSHD and control myoblasts throughout 4q35 and 10q26. Moreover, our FSHD myoblast cell strains, which are grown under optimal conditions and contain >85% desmin-positive cells, grew and differentiated just as efficiently as control myoblasts, gave normal-looking myotubes, and had no deficiency in *MYOD1* transcription although several hundred genes were dysregulated in FSHD vs. control myoblasts [2]. Retention of myogenesis together with preservation of a wild-type myoblast replication timing profile suggests that the large-scale myogenesis-specific chromatin reorganization events are normal in FSHD myoblasts.

### No significant replication timing differences exist genome-wide in myoblasts from FSHD vs. control individuals

**Figure 5:**
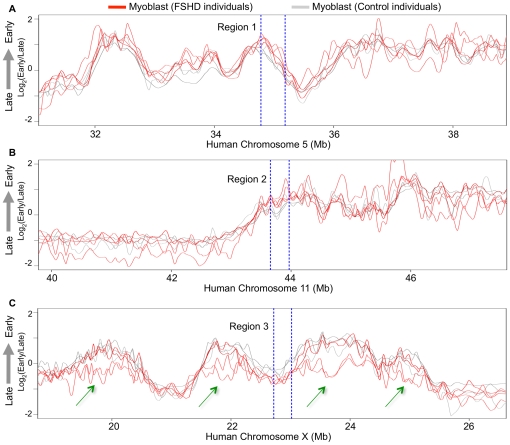
Regions of greatest statistical difference between control and FSHD myoblast replication timing profiles. (A, B, C) Primary human myoblast replication timing profiles are shown for 3 control (gray) individuals and 4 FSHD (red) patients across the top three 200 kb regions (on chromosomes 5, 11, and X respectively) of greatest difference as identified using a distance-maximizing statistical method [39]. Each 200 kb region of greatest difference between FSHD and control myoblasts is flanked by dashed, blue lines. Green arrows in panel C mark regions with gender-dependent differences caused by X-chromosome inactivation. The two lower curves in these regions are from the two female-derived myoblast samples and the other curves are myoblasts from males.

Next, to locate any regions with consistent replication timing changes in FSHD profiles, we employed a statistical method that selects 200 kb windows in which replication timing differences are both minimized between groups of designated replicates and maximized between designated test groups [39]. This statistical algorithm identified very few small differences between all control versus all FSHD replicate profiles. Figure 5 compares the replication profiles from normal and FSHD myoblasts across 8 Mb surrounding each of the three most significant differences detected by our algorithm. Even these selected regions were highly similar in all replicates, indicating that the FSHD disease background had no detectable effect on genome-wide replication timing in myoblasts.

### Individual myoblast timing profiles are indistinguishable except across regions affected by X chromosome inactivation

**Figure 6:**
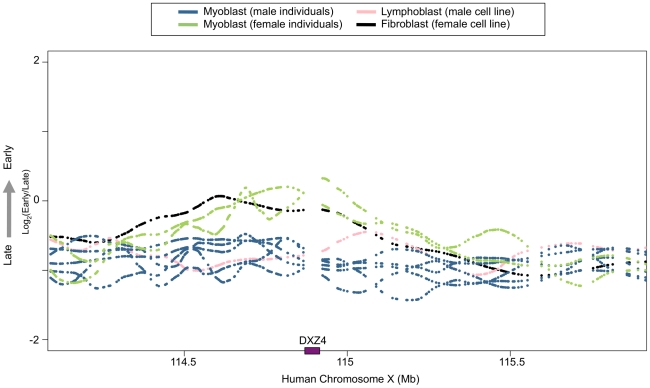
The region containing DXZ4 replicates early on the inactive X chromosome. Primary human myoblast replication timing profiles are shown for 2 female (green) and 5 male (blue) individuals across the DXZ4 locus on chromosome X. Profiles for male lymphoblastoid (pink) and female fetal lung fibroblast (black) cell lines are also shown. Gaps in each profile represent regions between microarray probes. The purple box on the x-axis marks the position of DXZ4.

To determine whether any replication timing differences could be detected between genetically polymorphic individuals and to determine the sensitivity of detection of modest average replication timing differences, we examined replication timing of the X and Y chromosomes in individual myoblast profiles. Although no autosomal regions exhibited differences, female profiles were easily distinguished from male samples, both by the absence of hybridization to Y-chromosome specific probes on the microarray, and also by tendency toward later average replication timing across the X chromosome (Figure 5C). This is in accord with these regions replicating later on one of the two female X chromosomes after X inactivation [40]–[42]. These results confirm that the replication-timing program is conserved across polymorphic human individuals and also demonstrates that significant monoallelic replication-timing differences can be detected by our genome-wide profiling method. This is consistent with our previous demonstration that the method used here can readily detect replication-timing differences between female mouse cells containing two active X chromosomes versus one active and one inactive X chromosome (Xi) [26]. Intriguingly, all the replication-timing differences involved switches to later replication except in the region containing the DXZ4 repeat, which was earlier on the Xi (Figure 6). Early replication of DXZ4 on the Xi is consistent with the array's euchromatic structure and CCCTC binding factor (CTCF) association specifically on the Xi [43]–[46].

### Myoblast replication timing is most similar to fibroblast replication timing

The genome-wide profiles from this study also revealed that human myoblast replication timing profiles most closely resemble fibroblast timing profiles amongst the cell types compared (Figure 2B), as was previously reported for mouse fetal myoblasts and embryonic fibroblast lines [26]. The second closest relationship in replication timing was seen for myoblasts and ESC-derived mesoderm cells, suggesting a common developmental origin. Since the profiled fibroblasts and myoblasts both underwent *in vivo* differentiation prior to isolation whereas all other cell types examined were differentiated *in vitro* from embryonic stem cells, it is possible that this similarity is an artifact of cell culture methods. Primary smooth muscle cells have been shown to dedifferentiate in culture to take on a fibroblast-like phenotype [47], suggesting that at least some muscle types are at a differentiation state highly similar to that of fibroblasts and demonstrating that culture methods can influence the differentiation state. Further complicating matters, fibroblast cell strains are also known to comprise a variety of cell phenotypes [48], [49]. Thus, more precise fibroblast classification methods and timing profiles from other primary cell types are needed to discern whether the observed similarity between myoblast and fibroblast timing profiles is due to common developmental origins or artifacts of *in vitro* culture and differentiation.

### Conclusions

In summary, we report the first genome-wide replication timing study in primary human cultures. The lack of differences between control and FSHD myoblasts in their DNA replication profiles suggests that most major long-range chromatin organizational events during myoblast formation proceed normally in FSHD patients. Our findings also underscore the cell type-specificity and reproducibility of genome-wide replication timing profiles in the FSHD disease background between polymorphic primary cell lines of the same cell type. Moreover, we found that human cells display the same high similarity between myoblast and fibroblast genome-wide replication timing profiles that was observed in mice.

## Materials and Methods

### Ethics Statement

This research was reviewed and approved by a Florida State University Human Subjects Committee based on Institutional Review Board approval at the Tulane Health Science Center and the University of Rochester School of Medicine and Dentistry and the University of Mississippi Medical Center in Jackson where duly signed patient consent forms were obtained and actual sample collection was performed.

### Myoblast isolation and cell culture

Myoblasts cultures (Table 1) were generated from muscle biopsies from three normal-controls, CM1 (42 Y, M), CM4 (27 Y, M), and CM5 (31 Y, M), and four FSHD patients, FM1 (41 Y, M), FM01 (45 Y, F), FM5 (29 Y, M), and FM7 (18 Y, F). The muscle samples used for generating myoblast cultures were quadriceps except for FM7 and CM5, which were from deltoid muscle and an unknown surgical sample, respectively. The FSHD biopsies were all from moderately affected tissue. FSHD samples had 3 (FM5 and FM7) or 6 (FM1 and FM01) copies of the D4Z4 repeat unit in the contracted, pathogenic D4Z4 array at 4q35. D4Z4 array sizes at 10q26 were known for three samples as follows; FM01 had one contracted array with 7 copies of the D4Z4 repeat unit in addition to the contracted 4q35 array and FM7 and CM1 had all D4Z4 arrays with more than 15 copies. Myoblasts were propagated as previously described [2], [18]. The myoblast cultures at passage 9 used for this study contained >85% myoblasts as determined by desmin immunostaining.

**Table 1:** Myoblast culture source information.

ID	Age	Gender	Biopsy Site
FM1	41	M	Quadriceps
FM5	29	M	Quadriceps
FM01	45	F	Quadriceps
FM7	18	F	Deltoid
CM1	42	M	Quadriceps
CM4	27	M	Quadriceps
CM5	31	M	Unknown skeletal muscle surgical sample

Lymphoblast [male lymphoblastoid cell line with normal (46, XY) karyotype, CO202 ECCAC no. 94060845], ESC (BG01, BG02, H7, H9), BG01-derived NPC, and ESC-derived mesendoderm, mesoderm, and definitive endoderm replication timing datasets were previously published [27], [39]. Unpublished fibroblast [female fetal lung, IMR90 (T. Chandra et al., unpublished)] is included with permission. Differentiation of BG02 ESCs to mesendoderm (DE2) and definitive endoderm (DE4) was performed by switching from defined media [50] to DMEM/F12 supplemented with 100 ng/ml Activin A and 20 ng/ml Fgf2 for two and four days, respectively, with 25 ng/ml Wnt3a added on the first day. Mesoderm was derived by adding 100 ng/ml BMP4 to DE2 cells.

### Genome-wide replication timing profile generation

Genome-wide replication timing profiles from passage 9 myoblasts were generated and analyzed as described [23], [35] using a Human whole-genome triplex microarray with one probe every 2.5 kb (Roche NimbleGen Inc., 090210_HG18_WG_CGH_v3.1_HX3; 719,690 oligonucleotide probes). Sample labeling, microarray hybridization and data extraction were performed according to standard procedures recommended by NimbleGen. A complete replication-timing dataset for all probes is downloadable and graphically displayed at http://www.replicationdomain.org [51]. In compliance with MIAME guidelines, all microarray data has also been deposited in the GEO database (Accession numbers pending manuscript acceptance).

### Replication timing profile analysis

Replication timing datasets (Table S1) were normalized and scaled together using the limma package in R as previously described [23], [35]. Loess smoothing was applied across a span of 300kb to normalized replication timing ratios (log_2_early/late) at each probe to generate a genome-wide profile. For clustering and identification of differentially replicated segments, timing values were averaged in windows of approximately 200kb. Regions with consistent changes in replication timing in FSHD myoblasts were identified using a Monte Carlo algorithm [39] and custom R/Bioconductor scripts.

## Supporting Information

Table S1 **Standard deviations, means and medians are listed for all replication timing profile datasets (Sample ID) used in this study.**
(DOC)
