# Correction: DNA Replication Timing Is Maintained Genome-Wide in Primary Human Myoblasts Independent of D4Z4 Contraction in FSH Muscular Dystrophy

**DOI:** 10.1371/annotation/4fc6a867-640c-4454-ac3b-eb8a386d0ec4

**Published:** 2012-01-12

**Authors:** Benjamin D. Pope, Koji Tsumagari, Dana Battaglia, Tyrone Ryba, Ichiro Hiratani, Melanie Ehrlich, David M. Gilbert

The GEO study accession number was not noted in the published article. The GEO study accession number is GSE34197.

There was an error in the funding statement. The correct funding statement is: This work was funded by National Institutes of Health grants GM083337 to DMG and NS048859 to ME, and a grant from the FSHD Global Research Foundation to ME. The funders had no role in study design, data collection and analysis, decision to publish, or preparation of the manuscript. 

