# Supplementary material for: DNA Replication Timing Is Maintained Genome-Wide in Primary Human Myoblasts Independent of D4Z4 Contraction in FSH Muscular Dystrophy
Source: PLoS One. 2011 Nov 11;6(11):e27413. doi: 10.1371/journal.pone.0027413 (PMC3214052; doi:10.1371/journal.pone.0027413)
Supplement: Table S1 — Standard deviations, means and medians are listed for all replication timing profile datasets (Sample ID) used in this study. (DOC) [file pone.0027413.s001.doc]

Table S1 Replication profile dataset statistics

| Sample ID | Standard deviation | Mean | Median |
| --- | --- | --- | --- |
| FM1 | 0.803 | 0.026 | 0.021 |
| FM5 | 0.829 | 0.015 | 0.020 |
| FM01 | 0.867 | 0.014 | 0.139 |
| FM7 | 1.000 | -0.006 | 0.116 |
| FSHD Average | 0.831 | 0.011 | 0.137 |
| CM1 | 0.755 | 0.003 | -0.044 |
| CM4 | 0.875 | 0.010 | 0.074 |
| CM5 | 0.810 | 0.012 | 0.069 |
| Control Average | 0.789 | 0.008 | 0.038 |
| Fibroblast | 0.804 | 0.025 | -0.149 |
| Mesoderm | 0.903 | 0.042 | -0.118 |
| Neural Progenitor | 0.809 | 0.064 | 0.062 |
| Lymphoblast | 0.859 | 0.034 | -0.217 |
| Embryonic Stem Cell | 0.917 | 0.086 | -0.041 |
| Mesendoderm | 0.903 | 0.042 | -0.118 |
| Definitive Endoderm | 0.891 | 0.010 | -0.108 |
